# Exercise and the Aging Endothelium

**DOI:** 10.1155/2013/789607

**Published:** 2013-08-01

**Authors:** Saeid Golbidi, Ismail Laher

**Affiliations:** Department of Pharmacology and Therapeutics, Faculty of Medicine, University of British Columbia, Vancouver, BC, Canada V6T 1Z3

## Abstract

The endothelium plays a critical role in the maintenance of cardiovascular health by producing nitric oxide and other vasoactive materials. Aging is associated with a gradual decline in this functional aspect of endothelial regulation of cardiovascular homeostasis. Indeed, age is an independent risk factor for cardiovascular diseases and is in part an important factor in the increased exponential mortality rates from vascular disease such as myocardial infarction and stroke that occurs in the ageing population. There are a number of mechanisms suggested to explain age-related endothelial dysfunction. However, recent scientific studies have advanced the notion of oxidative stress and inflammation as the two major risk factors underlying aging and age-related diseases. Regular physical activity, known to have a favorable effect on cardiovascular health, can also improve the function of the ageing endothelium by modulating oxidative stress and inflammatory processes, as we discuss in this paper.

## 1. Introduction 

The global population, especially those in developed countries, is getting older and this trend is predicted to continue in the coming decades [1, 2]. Some have defined aging as a decreased ability to resist cellular stresses or insults [3, 4], and in fact, aging is one of the most important cardiovascular risk factors for predisposing conditions such as diabetes, hypertension, and hypercholesterolemia. Accordingly, the incidence and prevalence of clinical and subclinical cardiovascular diseases increase dramatically with age [2], making cardiovascular disease the most common cause of death among the elderly.

The endothelium has a primary role in adjusting vascular function by the production of nitric oxide (NO) and other biologically active vasodilator materials [5] that decrease vascular resistance, inhibit platelet adhesion and aggregation, and decrease vascular smooth muscle cell proliferation. Alterations in the control of these processes, a feature of endothelial dysfunction, often leads to atherosclerosis and other vascular disorders [6] that are accompanied by a proinflammatory, proliferative, and procoagulatory state [7]. The endothelium is ideally placed to bear the brunt of hemodynamic stresses, oxidized lipids, and oxidative radicals, all of which increase their vulnerability to aging [8]. 

Chronic aerobic exercise improves cardiovascular function in humans. This is true not only in healthy subjects without underlying risk factors [9], but also in older people [10] and those with cardiovascular risk factors [11]. Indeed, those with cardiovascular risk factor/disease will benefit more. For instance, eight weeks of exercise significantly improve endothelial function, as measured by flow-mediated dilation, in diabetic patients [12] but not in healthy subjects [13]. 

Although there are clear health benefits of exercise in the elderly, a detailed understanding of the molecular basis underlying these improvements remains incomplete. In this minireview, we discuss some mechanisms thought to be involved in endothelial aging. We focus on the role of oxidative stress and subsequent inflammation and the role of exercise in boosting antioxidative and anti-inflammatory mechanisms. 

## 2. Endothelial Function

The endothelial cells form a monolayer that lines blood vessels to form an interface between circulating blood and the smooth muscle layer. In addition to its barrier function, the endothelium modulates coagulation, growth, and inflammation throughout the circulatory system. It also contributes to adjusting tissue perfusion by secreting several vasoactive substances [14], which can be vasoconstrictors (endothelin-1, angiotensin II, thromboxane A2, etc.) or vasodilators (NO, prostacyclin, and endothelium-derived hyperpolarizing factor (EDHF), etc.) [15]. Among the many vasoactive agents released by the endothelium, NO has been characterized in greatest detail. It is released in response to a variety of chemical and physical stimuli to cause vasodilation, such that one of the most common methods for assessing endothelial health is by determining vascular capacity to produce NO [16]. NO is produced by the catalytic activity of NO synthase (NOS), which transforms L-arginine to L-citrulline. All isoforms of NOS require five cofactors/prosthetic groups: flavin adenine dinucleotide (FAD), flavin mononucleotide (FMN), heme, tetrahydrobiopterin (BH4), and Ca^2+^/calmodulin. Calcium is required for the activation of neuronal NOS (nNOS) and endothelial NOS (eNOS) but not for the activity of inducible NOS (iNOS) [17]. eNOS binds to caveolin-1 in endothelial cell caveolae, a subset of specialized lipid domains that form invaginations and so increase intracellular microdomains where organelles and anchoring proteins aggregate. Caveolin-1 inhibits eNOS activity, and this interaction is regulated by Ca^2+^/calmodulin [18]. Upon agonist activation, the increases in intracellular calcium results in Ca^2+^/calmodulin binding, which then displaces caveolin and reverses its inhibitory interaction with eNOS [19]. Mechanical stimuli such as shear stress and vascular smooth muscle stretch also raise intracellular calcium concentrations. Several chemical events such as interaction with Ca^2+^/calmodulin, heat shock protein 90, and subsequent association of Akt results in eNOS phosphorylation at Ser1177 are involved in NO production [19]. Released NO causes vasorelaxation, which in turn results in increased blood flow and reduced blood pressure, inhibition of platelet adhesion and aggregation, inhibition of leukocyte adhesion, reduction in smooth muscle proliferation, and retardation of atherogenesis [4].

## 3. Endothelial Dysfunction and Aging

Endothelial dysfunction is defined as functional alterations in endothelial physiology characterized by reduction of vasodilator substance output (in particular NO) and augmentation in endothelium-derived contracting factors [4]. This imbalance leads to a vasoconstrictive, hypercoagulative, proliferative, and proinflammatory state, so favoring atherosclerosis [7]. Over time, most humans are exposed to a variety of modifiable cardiovascular risk factors, such as hyperglycemia and insulin resistance, obesity, altered lipid profile, hypertension, and glomerulosclerosis. All these confounding factors get exacerbated with age-related decreases in physical activity. Even short periods of inactivity lead to insulin resistance and endothelial dysfunction [35–37]. Bed rest of about 48 hours induces vascular dysfunction, which is then followed by insulin resistance, dyslipidemia, and increased blood pressure [38]. Thus, shortening of hospital stays and bed confinement periods are highly beneficial in the elderly. Bed rest also increases circulating endothelial cells [39], possibly due to increased endothelial cell apoptosis resulting from reduced shear stress during bed rest. These stresses activate endothelial repair systems. According to Thorin et al., as long as the damage is minimal or maintenance systems work properly as in young subjects, the functional capacity of the endothelium is preserved. In cases of severe damage, injured cells are omitted by a poorly described mechanism and replaced by dividing neighboring cells. Circulating progenitor endothelial cells also play a role in the repair mechanisms of injured endothelial cells. However, as part of the aging processes, the cumulative effects of stresses coupled with the inevitable metabolic changes that occur with time, there is a decline in the function and repair capacity of the endothelium. Since endothelial cells can only undergo a limited number of divisions, they eventually enter a state of senescence, which is an endogenous and hereditary process of biological aging, in which cells are still metabolically active, but express a pro-inflammatory, prooxidative, and proatherogenic phenotype [40]. The cumulative effects of these parameters strongly promote a decline in the functional capacity of endothelium. All forms of cardiovascular disease have an increased prevalence in the elderly, even in those free of cardiovascular risk factors [41]. Since there are no changes in endothelium-independent vasodilation in older humans and animals, it is reasonable to suggest that vascular age-dependent endothelial changes largely reflect the release NO [15], although there are also some changes in the production and release of other endothelial derived vasodilators such as prostacyclin and EDHF, along with increases in vasoconstrictor prostanoids [42]. There are also several age-related structural changes in endothelium such as increases in the expression of adhesion molecules, permeability, sensitivity to apoptotic stimuli, with decreases in angiogenic and regenerative capacities [43, 44].

## 4. Mechanisms of Endothelial Aging

There are a number of mechanisms proposed to explain age-related endothelial function. However, oxidative stress and inflammation appear critical to this process.

### 4.1. Oxidative Stress and Endothelial Dysfunction

Oxidative stress is an imbalance between production of oxidizing agents, such as free radicals, and opposing antioxidant systems which scavenge or metabolize those reactive agents. Free radicals are reactive chemical molecules having a single unpaired electron in an outer orbit. This unstable configuration provides energy which is released through reactions with adjacent molecules such as proteins, lipids, carbohydrates, and nucleic acids. The majority of free radicals that damage biological systems are oxygen-free radicals [45]. Oxygen-free radicals or, more generally, reactive oxygen species (ROS), as well as reactive nitrogen species (RNS), are products of normal cellular metabolism. Oxidative stress interferes with endothelial function in different ways, but the most prominent mechanism is via reduction of NO bioavailability, which is the net product of the rate of NO production and its degradation by superoxide [46]. 

In the case of NO generation, reduced vasoconstrictive responses to the NOS inhibitor N(G)-monomethyl-L-arginine (L-NMMA) in older patients [47] and reduced shear stress-induced NO release and vasodilation in older animals [48] suggest decreased production of NO in aged endothelium. In spite of these, direct measurements of eNOS in aged animals were inconclusive, as raised, fallen, or unchanged levels have been reported [49–52]. 

The increased production of superoxide anions in the aging vascular wall rapidly inactivates NO [53, 54]. Cyclooxygenase (COX) and NADPH-oxidase have central roles in ROS production [47, 55–58]. Removal of the endothelium or inhibition of NADPH-oxidase reduces vascular superoxide generation in the aorta of aged Wistar-Kyoto rats [55]. Superoxide rapidly reacts with NO to generate cytotoxic peroxynitrite (ONOO^−^), a reaction with several consequences. First, ONOO^−^ alters the function of biomolecules by protein nitration as well as by causing lipid peroxidation [59]. For example, potassium channels, which hyperpolarize vascular cells and mediate regulate vasorelaxation, are inhibited by nitration [60, 61]. Second, ONOO^−^ causes single-strand DNA breakage, which in turn activates nuclear enzyme poly(ADP-ribose) polymerase (PARP) (a nuclear DNA-repair enzyme) [62]. Third, it decreases NO bioavailability causing impaired relaxation and inhibition of the antiproliferative effects of NO. Furthermore, ONOO^−^  oxidizes BH4, an important cofactor for NOS, leading to the uncoupling of eNOS and causing it to produce superoxide instead of NO. ROS-induced peroxidation of membrane lipids alters the structure and the fluidity of biological membranes, so having global detrimental effects on vascular function [53]. The role of oxidative stress in aged endothelial dysfunction is shown by the ability of vitamin C to restore the impaired vasodilatory response to acetylcholine only in subjects aged 60 years or older [47]. This indicates that oxidative stress is a critical mechanism for endothelial dysfunction only in older subjects. Administration of BH4 also improves flow-mediated dilatation (FMD) in older sedentary subjects, while having no beneficial effects in young or older trained people [63].

Antioxidant deficiency is another mechanism for oxidative stress in endothelial cells. All cells have evolved highly complex enzymatic and nonenzymatic antioxidant systems that act synergistically to defend the body from free radical-induced damage. The most efficient enzymatic antioxidants are glutathione peroxidase, catalase, superoxide dismutase, heme oxygenase-1 (HO-1), NAD(P)H quinone oxidoreductase-1 (NQO-1), and thioredoxin. Nonenzymatic antioxidants include vitamins E and C, thiol antioxidants (glutathione, thioredoxin) [64]. Attenuation of antioxidant defense mechanisms during the aging process has been proposed in some studies. For instance, reduced concentration of plasma SOD, but not cellular SOD, occurs in rats [65]. Higher production of peroxynitrite causes antioxidant enzyme deactivation, as is the case for manganese SOD (MnSOD) in mitochondria [51]. Levels of protein expression and enzymatic activity of glutathione peroxidase 1 (GPX-1) are lower in proangiogenic endothelial progenitor cells (derived from cultured blood mononuclear cells) from older subjects [66].

### 4.2. Inflammation and Endothelial Dysfunction

Inflammation has a prominent role in the pathogenesis of several cardiovascular diseases. Atherosclerosis is an inflammatory disease that is mediated by monocyte-derived macrophages which accumulate in arterial plaques and become activated to release cytokines that cause tissue damage [67]. As evidence accumulates favoring the role of inflammation during the different phases of atherosclerosis, it is likely that markers of inflammation such as high sensitivity C-reactive protein (hs-CRP) could be increasingly used to provide additional insights on the biological status of atherosclerotic lesions. Elevations of CRP are considered independent predictors of cardiovascular events and of the outcome of acute coronary syndromes [68]. Besides their roles markers of systemic inflammation and as predictors of cardiovascular risk, CRP and other inflammatory cytokines also directly trigger vascular dysfunction [69], possibly by altering calcium channel expression and activity [70], upregulating of Rho-kinase expression and function [71], increasing ROS production [72], and/or enhancing COX expression [73]. In turn, COX-derived constrictor prostanoid(s) products cause vascular hypercontractility [74, 75] and increased formation of ROS [76]. Increases in COX-induced ROS production and the expression of proinflammatory mediators, such as IL-1B, IL-6, TNF-*α*, COX-2, and iNOS, occur during aging [77].

The NF-*κ*B pathway is a critical component of inflammatory processes activated by oxidative stress [33, 78]. NF-*κ*B is an ubiquitous transcription factor with multiple roles such as mediating inflammatory responses to a variety of signals, immune function, endothelial cell activation, and control of cell growth [79–81]. NF-*κ*B is normally located in the cytoplasm in an inactive form by virtue of binding to a family of inhibitory NF-*κ*B (I*κ*B) proteins. Upon cell stimulation by a wide variety of stimuli, signals responsive IKK-*α* and IKK-*β* (also known as IKK-1 and IKK-2) are activated, which results in the phosphorylation of I*κ*B and its proteasomal degradation. I*κ*B degradation liberates NF-*κ*B, allowing it to translocate to the nucleus and induce gene expression of a number of proinflammatory cytokines, such as IL-1*β*, IL-6, TNF-*α*, COX-2, lipoxygenase, iNOS, and adhesion molecules (VCAM-1, ICAM-1, PCAM, E-selectin). Aging increases NF-*κ*B levels due to activation of IKK*α*/*β* and degradation of I*κ*B [82]. Under usual conditions, the activation of NF-*κ*B during inflammation is temporary and limited. In aging, however, a chronic and self-perpetuating condition exists. Proteins such as TNF-*α*, IL-1, IL-6, and COX-2 that are NF-*κ*B induced also activate NF-*κ*B production, thus creating a vicious cycle [83]. Aging increases plasma levels of TNF-*α*, IL-6, IL-1*β*, CRP, and inflammatory blood cells [84–86]. The plasma concentration of IL-6 correlates with senile neural atrophy [87] and inflammatory diseases, such as type 2 diabetes and atherosclerosis [88]. Plasma levels of TNF-*α* and IL-6 are also predictors of disability and mortality among elderly [89]. 

## 5. Role of Exercise in Improving Endothelial Function in Elderly

The significance of exercise as a modifiable risk factor for cardiovascular disease is widely acknowledged [90]. Physical inactivity and poor diet, preceded only by tobacco, are the leading causes of death [91]. The American College of Sports Medicine, defines exercise as* “Any and all activity involving generation of force by the activated muscle(s) that results in disruption of a homeostatic state”* [92]. Exercise can be classified by the type, intensity, and duration of activity. Endurance exercise is characterized by prolonged and continuous periods of contractile activity (high repetition) against low resistance. Resistance exercise (also termed strength training) involves short periods of contractile activity (low repetition) against a high opposing resistance. Sprint exercise consists of short periods of maximal (intense) repetitive contractile activity with a low interval and against a low resistance, for example, running 100 m sprint race. However, sprint training can also be performed against high resistance resulting in a combination of resistance and endurance modalities, for example, running with added weights [93].

Increased physical activity and fitness, of both men and women, reduce the relative risk of death by 20–35% [94, 95]. Some studies even suggest greater benefits (up to 50% risk reduction) for exercise in terms of all-cause mortality and death from cardiovascular disease [96]. Brown et al. in almost one decade follow-up study evaluated the relationship between physical activity and risk of all-cause mortality in a large number of elderly (7080 women aged 70–75 and 11668 men aged 65–83) [97]. They found an inverse dose-response relationship between exercise and all-cause mortality. Risk reductions were 30–50% higher in females than in males in every category of exercise intensity. This study clearly shows that there are clear health benefits from all levels of physical activity. Regular aerobic exercise can slow down the age-related losses in endothelial function [98] supposedly by restoration of NO availability consequent to prevention of ROS production [99]. Aging is associated with a limited capacity of the vasculature to release NO, as older subjects show reduced levels of plasma nitrite in response to exercise [100]. The difference in plasma nitrate/nitrite ratio between older and young sedentary subjects is reduced by exercise. Heat-stimulated hand and foot skin increased blood flow are higher in trained older subjects compared to sedentary matched controls and are correlated with nitrate/nitrite ratios, suggesting better endothelial function secondary to greater NO bioavailability [101]. Trained elderly subjects also exhibit higher flow-mediated brachial artery dilation compared to sedentary counterparts [100].  Table 1 summarizes the findings of recent clinical studies on the endothelial benefits of exercise in the elderly.

### 5.1. Effect of Exercise in Mitigating Oxidative Stress

Exercise training upregulates antioxidant defense mechanisms in several tissues, presumably due to increased levels of oxidative stress that occurs during exercise. Exercise-induced production of ROS is proposed to evoke specific adaptations such as increasing repair mechanisms for oxidative damage, increasing resistance to oxidative stress, and lowering levels of oxidative damage. On the other hand, excessive production of ROS can have detrimental effects. Boosting levels of intrinsic antioxidant potential and reduction in lipid peroxidation occur in healthy elderly men after habitual physical activity [102].

A critical role has recently been described for a transcription factor “nuclear factor (erythroid-derived 2)-like 2 (Nrf2)” against oxidative stress. Normally, Nrf2 is located in the cytoplasm and kept dormant by the cytoplasmic repressor Kelch-like ECH-associated protein 1 (Keap1). A variety of activators, including oxidative free radicals, release, and translocate Nrf2 into the nucleus where it regulates the expression of antioxidant enzymes such as NQO-1, glutathione-S-transferase, glutathione peroxidase, and HO-1 [64]. Diminished Nrf2 activity contributes to increased oxidative stress and mitochondrial dysfunction leading to endothelial dysfunction, insulin resistance, and abnormal angiogenesis as observed in diabetics [103]. HO-1, which is mainly induced through the Nrf2-Keap1 signaling pathway (also known as heat shock protein 32), is the inducible isoform of heme oxygenase that catalyzes NADPH-dependent decomposition of heme to carbon monoxide (CO), ferrous iron, and biliverdin [104]. Three isoforms of HO have been identified: both HO-2 and HO-3 are 33-kDa constitutively expressed isoforms [105]. An important role of HO-1 in the antioxidant defense system arises from an induction of ferritin synthesis that diminishes the cellular pool of free iron [106] and also from the enhancement of bilirubin levels, which are potent antioxidants [107]. Carbon monoxide activates soluble guanylate cyclase, a key enzyme in the cell signaling cascade leading to relaxation of smooth muscle, and thrombocyte disaggregation. Carbon monoxide also affects cellular metabolism and counteracts proinflammatory cytokine cascades [105]. HO-1 is a sensitive and reliable marker of oxidative stress [108] and cytoplasmic expression levels of HO-1 increase in leukocytes of endurance-trained male subjects after a half-marathon run [109]. There is a paradoxical increased expression of HO-1 in a control group of untrained men at rest, suggesting that the downregulation of the baseline expression of HO-1 in athletes reflects an adaptation mechanism to regular exercise training [109]. The direct effect of exercise on Nrf2 expression has received much less attention except for a report that exercise increases nuclear levels of Nrf2 in the proximal renal tubules of old rats [110].

The increased expression of eNOS after exercise both in animals and human beings [111–114] also occurs in patients with stable coronary artery disease and chronic heart failure [115, 116]. Exercise-induced upregulation of vascular eNOS expression is closely related to the frequency and the intensity of physical forces within the vasculature, especially shear stress. Shear stress is the product of all the perpendicular and parallel flow-mediated forces on endothelial cells. The types of these hemodynamic forces, either laminar or oscillatory, greatly impact the function and properties of endothelial cells and also determine the signal transduction pathways that are activated. Laminar flow, which is augmented during moderate and intense physical activities, upregulates eNOS expression—while oscillatory forces, which are associated with hypertension, leads to increased NADPH-oxidase activity and augments oxidative stress [40]. The mechanotransduction mechanisms that sense physical forces to cause altered gene expression are not completely described. Some reports suggest that activation of inward rectifying K^+^ cannels, followed by stimulation of outwardly rectifying Cl^−^ channels, plays a major role in this process. Membrane hyperpolarization, due to inward K^+^currents, drives extracellular Ca^2+^ into the cells through two shear stress-dependent ion channels (P2X purinoceptors and transient receptor potential channels). Raised intracellular calcium levels lead to a dissociation of caveolae-bound eNOS and increased production of NO [117]. Other intracellular events are also thought to mediate increased NO production in response to shear stress; however, the relative importance of these mechanisms is not clear (Table 2). Increased NO synthesis secondary to amplified shear stress induces extracellular superoxide dismutase (SOD) expression in a positive feedback manner so as to inhibit the degradation of NO by ROS [34]. Another parallel mechanism that participates in this harmony is the upregulation of eNOS through exercise-induced ROS production, since exercise-induced increases in shear stress stimulates vascular production of ROS by an endothelium dependent pathway [118]. Endothelial NAD(P)H oxidase has a critical role in this process [119]. Superoxides are rapidly converted to H_2_O_2_ by SOD; hydrogen peroxide then diffuses through the vascular wall and increases the expression and activity of eNOS [120, 121]. Thus, increased expression of SOD1 and SOD3 (which facilitate the generation of H_2_O_2_ from superoxide) augments the effect of H_2_O_2_ on exercise-induced eNOS expression. On the other hand, eNOS expression is not increased in catalase overexpressing transgenic mice [112, 122]. Another putative mechanism is exercise-induced increases in arterial compliance which is mediated by reduction of plasma ET-1 concentration as well as the elimination of ET-1 mediated vascular tone. Twelve weeks of aerobic exercise training increase arterial compliance, while decreasing plasma ET-1 levels. Moreover, the increase in central arterial compliance observed with ET-receptor blockade before the exercise intervention was eliminated after exercise training [123]. These results indicate that endogenous ET-1 participates in the beneficial influence of regular aerobic exercise on central arterial compliance. 

### 5.2. Anti-Inflammatory Role of Exercise

Exercise produces a short-term inflammatory response that is accompanied by leukocytosis, increases in oxidative stress and plasma levels of CRP. This proinflammatory response is followed by a long-term anti-inflammatory adaptive response [124]. Regular exercise reduces CRP, IL-6, and TNF-*α* levels while increasing anti-inflammatory substances such as IL-4 and IL-10 [125, 126]. Controlling the release and activity of at least two cytokines, TNF-*α* and IL-6, could contribute to the natural protective effects of physical activity. Interleukine-6 (IL-6) is the first cytokine to be released into the circulation during exercise, and its levels increase in an exponential fashion in response to exercise [74]. Contracting skeletal muscles upregulate levels of IL-6 mRNA [127] and the transcriptional rate of the IL-6 gene is also markedly enhanced by exercise [128]. IL-6 acts as both a proinflammatory and anti-inflammatory cytokine: when secreted by T cells and macrophages, IL-6 stimulates the immune response and boosts inflammatory reactions, while muscle-produced IL-6 exerts anti-inflammatory effects through its inhibitory effects on TNF-*α* and IL-1*β*, and activation of interleukin-1 receptor antagonist (IL-1ra) and IL-10 [129]. IL-10 in turn reduces the production of several proinflammatory cytokines, such as TNF-*α* and IL-1*β* [130]. Exercise-induced increases in plasma IL-6 correlate with the muscle mass involved in exercise activity and the mode, duration, and intensity of exercise [131], and this is especially the case in older individuals [132]. Exercise also confers protection against TNF-induced insulin resistance [133]. In addition, Starkie et al. report that infusion of recombinant human IL-6 (rhIL-6) into human subjects simulated exercise induced an IL-6 response in the prevention of endotoxin-induced increase in plasma TNF-*α* [134]. Exercise also suppresses TNF-*α* production by an IL-6 independent pathway, as there are modest decreases in plasma TNF-*α* after exercise in IL-6 knockout mice [135]. Exercise-induced increases in epinephrine levels blunt the TNF-*α* response [136]. In addition, IL-6 enhances lipid turnover and stimulates lipolysis as well as fat oxidation via activation of AMP-activated protein kinase [137]. Mice deficient in IL-6 (IL6−/−) develop mature onset obesity and have disturbed carbohydrate and lipid metabolism that is partly reversed by IL-6 replacement. Other data indicate that centrally acting IL-6 exerts an antiobesity effect in rodents [138]. The lipolytic effect of IL-6 on fat metabolism was confirmed in two clinical studies of healthy and diabetic subjects [137, 139]. Visceral fat is potentially a cause of low-grade systemic inflammation, which in turn leads to insulin resistance, type 2 diabetes, and atherosclerosis [140]. During exercise, IL-6 also increases hepatic glucose production. Glucose ingestion during exercise reduces IL-6 production by muscles, suggesting that IL-6 is released by a reduction in glycogen levels during endurance exercise and the consequent adrenergic stimulation of IL-6 gene transcription via protein kinase A activation [141].

Physical activity also increases the expression of IL-15 in the skeletal muscles. This cytokine exerts anabolic effects in muscles by inducing protein synthesis and inhibiting protein degradation [130, 142]. In some animal studies, this cytokine prevents muscle wasting by attenuating apoptotic DNA fragmentation and downregulating TNF-driven apoptotic pathways [143]. In agreement with this, four weeks of exercise reduce the extent of TNF-triggered myocyte apoptosis in old rats [144, 145] 

## 6. Summary

Aging is an important independent risk factor for the development of cardiovascular disease, which is manifest as endothelial dysfunction. A large body of evidence underlines the importance of oxidative stress and inflammation as prominent features of the aging process. Reduced NO bioavailability and decreased responsiveness to other endothelial-derived vasodilators promote a vasoconstrictive, hypercoagulative, and proliferative state which favors the development of atherosclerosis. Vascular oxidative stress is the product of increased reactive oxygen species, such as superoxides, and reduced antioxidant defense. Oxidative stress-induced damage promotes a chronic inflammatory state which perpetuates a vicious cycle of endothelial dysfunction. Exercise training prevents and restores age-related impairment of endothelial function, possibly by the restoration of NO availability consequent to prevention of oxidative stress and alleviating inflammatory processes. 

## Figures and Tables

**Table 1:** Selected recent clinical trials (last 5 years) about the effects of exercise in elderly.

References	Patient groups and characteristics	Intervention and follow-up	Measured parameters	Outcome
[20]	(i) 57 subjects with a mean age of 65.6 ± 3.8 y divided to: (ii) Control placebo (iii) Resistance training (RT) (iv) Vit C/E supplementation (VS) (v) RT + VS	(i) RT performed 3 times a week for 6 months. (ii) Vit supplement was 1000 mg Vit C + Vit E 400 IU daily for 6 month	(i) Oxidative stress status and metabolic and lipid profile were determined at baseline and after 6 months. (ii) Fat mass and fat-free mass measured by DXA (iii) Muscle strength	After 6 months: (i) No difference in muscle strength (ii) RT + VS had a positive effect on the plasma antioxidant profile but not on the prooxidant status

[21]	(i) 34 healthy, obese, older women (55–79 y old) with mild to moderate physical impairments divided to into the following groups for 24 weeks: (ii) Weight loss plus exercise (WL + E) (iii) Educational control	(i) WL + E was weight management sessions + 3 supervised exercise sessions/w (ii) Educational group had monthly health lectures	(i) Body weight (ii) Walk speed (iii) Short physical performance battery (SPPB) (iv) Knee extension isokinetic strength	(i) WL + E lost more weight and walking speed increased significantly. (ii) SPPB improved in both groups with significant differences between groups.

[22]	(i) Peripheral blood mononuclear cells (PBMC) from 25 young adult (18–33 y old) and 40 older subjects (50–76 y old)	(i) 2 months of aerobic exercise (brisk walking 6 days/w, 50 min/day, 70% of maximal HR)	(i) mRNA expression of NF-*κ*B, receptor for AGEs (ii) Proinflammatory cytokines including TNF-*α*, MCP-1, NADPH-oxidase, iNOS	(i) In older subjects VO_2_ max and exercise time were increased (ii) Expression of proinflammatory genes was decreased

[23]	(i) 173 overweight or obese, postmenopausal, sedentary women randomized to: (ii) Aerobic exercise intervention (iii) Stretching control group for 12 months.	(i) Exercise intervention was 60–75% of maximal HR for ≥ 45 min per day, 5 days/w	(i) F_2_-isoprostane, VO_2_ max, body weight, body fat percentage, waist circumference, intra-abdominal fat surface area	(i) VO_2_ max increased and body weight decreased in exercise group. (ii) F_2_-isoprostane decreased in exercise group and increased in control group.

[24]	(i) Six older (71 ± 2 y) healthy men with mild hypertension	All subjects received the antioxidant cocktail and placebo in a double blind, balanced, crossover design and participated in the exercise protocol.	(i) Plasma free radical concentrations were verified via EPR spectroscopy (ii) Endothelial function was evaluated via FMD	(i) Prior to training, acute antioxidant exposure did not change resting BP or FMD. Six weeks exercise reduced BP. (ii) Antioxidant administration after exercise negated improvements.

[25]	Patients with IGT and CAD were randomly assigned to: (i) Exercise training (n = 13) (ii) Rosiglitazone (8 mg, n = 11) (iii) Control group (*n* = 10)	Exercise training consisted of 6 × 15 min/d in the 1st week followed by 30 min/d submaximal ergometer for 3 weeks	(i) FBS, lipid profile, HbA1c (ii) CRP, fibrinogen (iii) BMI (iv) FMD	(i) Triglycerides and uric acid decreased in exercise group (ii) FBS, HbA1c, LDL, HDL, CRP, fibrinogen, and BMI did not differ between groups (iii) In the exercise group, exercise capacity and FMD increased significantly

[26]	(i) 14 young subjects (25.7 ± 5.4 y) (ii) 13 older people (65.6 ± 10.7 y)	(i) 30 min of dynamic handgrip exercise at a moderate intensity	(i) Brachial artery diameter and blood flow were measured by Doppler ultrasound (ii) vWF was measured before, immediately and 30 min after exercise	(i) The change in plasma vWF was linearly correlated with the increase in shear stress during exercise in older individuals, but not in the young subjects.

[27]	(i) EPCs from elderly (*n* = 25, 67.8 ± 3.38 years) and young men (*n* = 22, 26.3 ± 3.15 y)	(i) 12 weeks of physical exercise	(i) In vitro endothelial function and in vivo reendothelialization capacity of EPCs (ii) Expression of CXCR4 and JAK-2 were measured	(i) In vitro function and in vivo reendothelialization capacity were reduced in elderly (ii) Exercise increased CXCR4 protein expression and JAK-2 phosphorylation

[28]	(i) 11 middle-aged/older men (ii) 15 postmenopausal women	(i) 8 weeks of brisk walking (6 days/w, 50 min/d)	(i) FMD	(i) FMD increased >50% in men but did not change in postmenopausal women

[29]	(i) 13 young men (27 ± 1 y) (ii) 15 older men (62 ± 2 y)	(i) 3-month aerobic exercise intervention in older subjects	(i) FBF was measured in response to ET-1 and selective (BQ-123) and nonselective (BQ-788) ET-1 inhibitors	(i) Vasoconstrictor response to ET-1 was blunted in older subjects (ii) BQ-123 increase FBF in the older subjects (iii) After 3-month exercise, vasoconstrictor responses to ET-1 increased in older people, while BQ-123 added modestly to this response

[30]	31 patients with type 2 diabetes and metabolic syndrome (mean age = 58 ± 6 years) were divided to: (i) High-intensity exercise (*n* = 10) (ii) Low-intensity exercise (*n* = 10) (iii) Controls (*n* = 11)	(i) 6 weeks of training	(i) Endothelial function examined by a high resolution ultrasound of the brachial artery, before and after 6 weeks training	(i) High intensity aerobic training improved endothelium dependent vasodilator response.

[31]	38 patients with type 2 diabetes divided to: (i) Exercise group (*n* = 21) (ii) Control (*n* = 17)	(i) Aerobic and resistance exercise for 3 months	(i) Endothelial function (FMD) (ii) Insulin resistance (iii) Adipocytokines (iv) Inflammatory markers (v) Incidence of CVE after 24 months	(i) HbA1c decreased in both groups (ii) FMD increased only in exercise group (iii) Incidence of CVE was higher in control group

[32]	209 patients with recent AMI divided to (i) Aerobic group (ET, *n* = 52, 56 ± 6 y) (ii) Resistance training (RT, *n* = 54, 57 ± 8 y) (iii) RT + ET (*N* = 53, 55 ± 9 y) (iv) No training (*n* = 50, 58 ± 7 y)	(i) 4 weeks of exercise training (ii) 1 month of detraining	(i) Endothelial function (FMD) (ii) vWF	(i) FMD increased in all 3 exercise groups independently of the type of exercise (ii) vWF decreased in all exercised groups (iii) Detraining returned FMD to baseline.

CRP: C reactive protein, CVE: cardiovascular events, EPR: electron paramagnetic resonance, FBF: forearm blood flow, FMD: flow-mediated dilation, HDL: high-density lipoprotein, HR: heart rate, IU: international unit, LDL: low-density lipoproteins, MCP-1: monocyte chemoattractant protein-1, NADPH-oxidase: nicotinamide adenine dinucleotide phosphate-oxidase, vit: vitamin, VO_2_ max= maximal oxygen consumption, vWF: von Willebrand factor, w: week, Y: years.

**Table 2:** Suggested shear stress sensing mechanisms by the endothelial cells and related intracellular signal transductions [33, 34].

Suggested candidates for sensing shear stress	Intracellular signal transduction
Cellular adhesion molecules	MAPK
Glycocalyx	Ras-ERK
Ion channels (K^+^, Cl^−^, Ca^2+^, P2X purinoceptor)	C-JNK
T K receptors	PI3- Kinase
GPCR	Akt
Caveola	FAK
Primary cillia	Rho Family GTPase
	NF-*κ*B
	PKC

C-JNK: c-jun N-terminal kinases, ERK: extracellular signal-regulated kinase, FAK: focal adhesion kinase, GPCR: G-Protein coupled receptors, GTP: guanosine triphosphate, MAPK: mitogen-activated protein Kinase, NF-*κ*B: nuclear factor-kappaB, PI3: phosphoinositide 3, PKC: protein kinase C, T K receptors: tyrosine kinase receptors.
